# Validity and Reliability of the Persian Version of the Dysphagia Handicap Index (DHI) 

**Published:** 2015-05

**Authors:** Faezeh Asadollahpour, Kowsar Baghban, Mozhgan Asadi

**Affiliations:** 1 *Health Promotion Research Center, Zahedan University of Medical Sciences, Zahedan, Iran. *; 2 *Department of Speech therapy, Hamadan University of Medical Sciences, Hamadan, Iran.*; 3 *Department of Speech Therapy, University of social welfare and rehabilitation sciences, Tehran, Iran.*

**Keywords:** Dysphagia, Handicap, Quality of life, Persian

## Abstract

**Introduction:**

The Dysphagia Handicap Index (DHI) is one of the instruments used for measuring a dysphagic patient’s self-assessment. In some ways, it reflects the patient’s quality of life. Although it has been recognized and widely applied in English speaking populations, it has not been used in its present forms in Persian speaking countries. The purpose of this study was to adapt a Persian version of the DHI and to evaluate its validity, consistency, and reliability in the Persian population with oropharyngeal dysphagia.

**Materials and Methods:**

Some stages for cross-cultural adaptation were performed, which consisted in translation, synthesis, back translation, review by an expert committee, and final proof reading. The generated Persian DHI was administered to 85 patients with oropharyngeal dysphagia and 89 control subjects at Zahedan city between May 2013 and August 2013. The patients and control subjects answered the same questionnaire 2 weeks later to verify the test-retest reliability. Internal consistency and test-retest reliability were evaluated. The results of the patients and the control group were compared.

**Results:**

The Persian DHI showed good internal consistency (Cronbach’s alpha coefficients range from 0.82 to 0.94). Also, good test-retest reliability was found for the total scores of the Persian DHI (r=0.89). There was a significant difference between the DHI scores of the control group and those of the oropharyngeal dysphagia group (P‹0.001).

**Conclusion:**

The Persian version of the DHI achieved Face and translation validity. This study demonstrated that the Persian DHI is a valid tool for self-assessment of the handicapping effects of dysphagia on the physical, functional, and emotional aspects of patient life and can be a useful tool for screening and treatment planning for the Persian-speaking dysphagic patients, regardless of the cause or the severity of the dysphagia.

## Introduction

Swallowing difficulties can be a symptom of many different disease processes and are associated with adverse health outcomes like malnutrition, dehydration, pneumonia, and death (1). Living with dysphagia brings physical, emotional, and social impacts and has direct consequences on patients’ quality of life (2-4,5-7). People with dysphagia are more likely to be anxious and depressed (8). Patients with dysphagia can be effectively evaluated and managed, particularly if the dysphagia is recognized before the development of medical complications such as aspiration pneumonia (9). Early detection through screening is an essential first step in the management to dysphagia (10). After being identified as being at risk of having dysphagia, further assessment of swallowing function is required. Videofluoroscopy (VFS) and fiberoptic endoscopic evaluation of swallowing (FEES) are mooted in literature to be the gold standards in the assessment of dysphagia. Another important step after screening is the completion of patient self-administered questionnaires (10). This tool can give an idea about how the patient perceives his/her swallowing problem and can be helpful in monitoring the patient’s prognosis (11). Healthcare professionals may have different perceptions of an individual's needs related to swallowing and may not consider or assess the nonphysical aspects of the disease. This may lead to dissatisfaction with healthcare. Professionals can address the psychosocial as well as the physical aspects of dysphagia by determining individuals' perspective of their needs.

Careful evaluation by the speech-language pathologist and other members of the dysphagia team with recommendations and a treatment plan formed jointly with the individual is recommended. Providing education about signs of dysphagia and changes in swallowing due to a disease process or treatment may improve QOL (12). 

Many quality of life questionnaires were developed for patients with dysphagia (11). Questionnaires on health-related quality of life with respect to oropharyngeal dysphagia can be found in the literature such as the SWAL-QOL, the MD Anderson Dysphagia Inventory (MDADI), and the Deglutition Handicap Index (DHI) (13-15). The SWAL-QOL is a symptom-specific outcome instrument that was developed to assess the severity of oropharyngeal dysphagia (13,16,17). This tool has been translated to other languages such as Chinese and Dutch (18,19). The instrument consists of 44 items and can be cumbersome to complete. Because of this limitation, the instrument has not been widely accepted in clinical practice (20). Other attempts to develop quality of life measures and/or symptom surveys that have focused on a certain subset of dysphagic patients (such as M.D. Anderson Dysphagia Inventory (MDADI) for head and neck cancer patients) are too cumbersome for clinicians to readily score and utilize expeditiously in the clinic (21). In many studies, MDADI was translated from English languages into Korean, Italian, Swedish Brazilian, and Dutch (14,21-24). The most common and convenient way to assess patient-reported outcome (PRO) is a self-report instrument as it is less time-consuming than interviews, guarantees that questions are asked in a standardized manner, and facilitates comparisons within and between groups. Self-report instruments can also be used in clinical practice for estimation of symptoms or treatment effects, to help patients communicate their problems and help health-care professionals to identify the major concerns of patients (19). The DHI study raises the issue of the scarcity of scales that evaluate dysphagia in a more complete way. It is noted that there are several assessment tools that analyze the symptoms of dysphagia, but much of them are specific to a single disease. Therefore, Silbergleit et al, developed the DHI (20). Development of the Persian Version of the DHI (P-DHI) will help physicians better understand the handicapped feelings of the Persian patient. This will facilitate the development of treatment strategies. Furthermore, the P-DHI could be used as a prognostic tool to monitor and document the effect of any traditional, pharmaceutical, or surgical therapeutic intervention that the patient receives. Currently, there is no Persian version of the DHI but its existence could significantly support the clinical practice of Persian-speaking patients with swallowing problems. Translation of questionnaire from one language to another raises many equivalence issues for cross cultural researchers, which are noticeable when target language has different dialects such as Persian. The aim of this study was to assess internal consistency, reliability, and validity of the P-DHI in Zahedan.

## Materials and Methods

*Subjects*

Eighty-five consecutive oropharyngeal dysphagia Iranian patients visiting the clinic at Zahedan University Hospital, between May 2013 and August 2013, were invited to participate in the study after consent. The group consisted of 45 males and 35 females. The mean age of patients was 61.8 years (range 37-80 years). The subjects represented a broad range of individuals with swallowing problems from a variety of medical diagnoses such as head and neck cancer, stroke, amyotrophic lateral sclerosis (ALS), Parkinson’s disease, and esophageal achalasia. To be included in the sample, a patient must have been diagnosed with oropharyngeal dysphagia by a laryngologist. Furthermore, the patient’s general condition must have been stable and the patient could not have any cognitive limitations. Patients were excluded if they did not speak Persian. The selected patients received verbal information about the study.

The control group consisted of 89 consenting Iranian normal adults, of which 42 were females and 47 were males. The mean age of this group was 64 years (range, 45-83 years). The subjects in the control group reported no history of dysphagia complaints or treatment for a swallowing disorder. 

*Questionnaire*

DHI is a patient-administered, 25 item questionnaire, in which the patient can assign three responses for each question (never, sometimes, and always), adding a value to each response (0,2 and 4, respectively) and reaching a score ranging from 0 to 100. Moreover, each patient performs a self-evaluation of their dysphagia, assigning a score from 0 (normal) to 7 (severe difficulty) (25). The DHI has 9 questions in the functional subscale, 9 question in the physical subscale, and 7 questions in the emotional subscale (20). The DHI was found to be a general application to a wide variety of individuals with swallowing disorders. The DHI may also be used with individuals with lower literacy levels and can be used in clinical and research settings alike (26). The original English version of the DHI (20) was translated into Persian by two translators who are proficient in both English and Persian. Two translations were synthesized into one interim version through the discussion between authors and translators. The interim version was translated into English again by a 3rd translator who was proficient in both English and Persian and this item was compared with the original items. The authors and translators gathered once again to check any potential errors. The Persian version of the DHI was then pilot-tested with 8 consenting Iranian subjects with oropharyngeal dysphagia. Subsequently, the P-DHI was amended according to their suggestions after reviewing the pilot data. Therefore, translation of DHI from English to Persian was performed with slight modifications to maintain semantic equivalence. For example, some words were substituted for other words with the same meaning in order to achieve better communication. The research team did not find it necessary to remove any of the 25 items from the original English version. Therefore, the Persian version was assembled with 25 questions arranged in three domains.

*Validation, testing, and statistical assessment*

Two independent and proficient translators judged all items of the P-DHI. The P-DHI was then administered to the dysphagia group and control group for them to fill it out without any assistance. 

The SPSS ver. 11.0 was used for statistical analysis. The correlation coefficient was used to measure the construct validity of P-DHI. The three subdomains were defined as the construct and the Spearman rho was calculated between subdomain scores and total scores to check whether the test scores measured the defined constructs well. The internal consistency of P-DHI was assessed using Cronbach’s alpha. The test–retest reliability was assessed by estimating the intra-class correlation coefficient.

## Results

The mean total P-DHI score for the control group and dysphagia group was 2.13 (SD=1.25) and 32.14 (SD=25.32) respectively. The mean scores of the three domains (functional, physical, and emotional) are represented (Table.1).

**Table 1:** A summary of the mean score for the functional, physical, and emotional domains and overall score in the subjects.

	**DHI domain (maximum possible score)**	**Mean**	**Median**
Control group	Functional (0-36)	0.14±0.60	2(0-4)
	Physical (0-36)	1.87±0.54	4(0-10)
	Emotional (0-28)	0.12±0.60	1(0-2)
	DHI total (0-100)	2.13±1.25	8(0-20)
Dysphagia group	Functional (0-36)	10.19±10.86	12(0-34)
	Physical (0-36)	15.23±9.79	11(0-33)
	Emotional (0-28)	6.53±5.76	4(0-28)
	DHI total (0-100)	32.14±25.32	28(0-92)
			

Among the dysphagia group, 13 patients rated their swallowing problem as being normal, 26 patients perceived their problem as mild, 37 patients perceived it as moderate, and 9 patients reported it as severe. Construct validity was checked by Spearman rho. The estimated correlation coefficient between the score of each domain and the total DHI score was significantly high (ranged from 0.802 to 0.927) (Table.2). 

**Table 2:** Spearman correlation coefficient between the score of each domain and total P-DHI score

**Domain**	**Total**	**Functional**	**Physical**	**Emotional**
Total DHI	1	-	-	-
Functional	0.8428*	1	-	-
Physical	0.802*	0.703*	1	-
Emotional	0.927*	0.829*	0.869*	1

* P<0.05.

The internal consistency was checked by Cronbach’s alpha in three subdomains. Overall internal consistency (α= 0.95) was excellent while for the three domains, the internal consistency ranged from 0.82 to 0.94 (Table.3). 

**Table 3:** Internal consistency of the P-DHI

**Subdomain**	**Number of questions**	**Cronbach’s coefficient for patients (n=85)**	**Cronbach’s coefficient for control (n=89)**
Emotional	7	0.85	0.82
Physical	9	0.94	0.79
Functional	9	0.82	0.78
Total	25	0.95	0.85
			

Fifty-eight of the 85 dysphagic patients and 60 of the control group patients completed the P-DHI twice over a period of 2 weeks. Excellent test–retest reliability was found for the total scores and for the P-DHI subscales. In addition, the intra class coefficients ranged from 0.849 to 0.890 (Table.4). The test–retest reliability for the functional domain was slightly higher than both the physical and emotional domains (Table.4). 

**Table 4:** Test-retest reliability of P-DHI

**Item**	**Intra class correlation coefficient**	
	Patient	Control
Emotional	0.875	0.82
Functional	0.885	0.83
Physical	0.849	0.78
Total	0.89	0.84
		

DHI scores showed a statistically significant difference between the patients and the control groups, for both overall DHI score and for each of the functional, physical, and emotional domains scores. (P‹0.001) (Table.5). 

**Table 5:** Comparison between dysphagia and control groups regarding domain score and total P-DHI score

**Group**		**N**	**Mean**	**P-value**
Functional	Patient	85	10.19	<0.001*
	Control	89	0.14	
Physical	Patient	85	15.23	<0.001*
	Control	89	1.87	
Emotional	Patient	85	6.53	<0.001*
	Control	89	0.12	
Total	Patient	85	32.14	<0.001*
	Control	89	2.13	

* Significance difference

## Discussion

A comprehensive evaluation of dysphagia should include not only some physiological measures (VFES and/or FEES), but also the patient’s perspective using Patient Reported Outcomes. There is a clear need to use validated questionnaires in the patients’ own language and which is also reflective of their culture and dialects. Therefore, the aim of this study was to contribute to the psychometric validation and reliability of the P-DHI in Zahedan in the self-administered dysphagia specific quality of life questionnaire. The results of the present study indicate that the P-DHI had strong internal consistency that was demonstrated in both the patients and the control groups. These results were similar to some of the studies done in the past (15,20,26). Test-retest reliability was calculated with a 2 week interval. Intra class correlation demonstrated good and significant stability in all subscales. These findings are similar to the findings of the original study by Silbergleit et al and to the studies of the Arabic versions of the DHI (20,26). The three domains of the P-DHI in the current study had also shown a strong internal consistency. In this study the dysphagic group had a slightly higher mean physical domain score as compared with the mean functional and emotional domain scores. Similar results have been reported in some of the studies in the past (15,26). This has been explained on the basis of a higher familiarity and association of the patients with the physical symptoms of dysphagia. This signifies that the physical domain of the P-DHI is the most prominent self-perceived parameter of dysphagia. In the current study, some items in the P-DHI have been modified to be better culturally adapted and understandable by Persian speakers in Zahedan. This has also been done in other studies such as Arabic and French DHI (15,26). This emphasizes the importance of the cultural adaptation of the DHI as some items, upon literal translation to another language, do not reflect the main idea behind the administered questions and may cause inappropriate responses by the patients.

## Conclusion

The P-DHI demonstrates psychometric values to allow its use in daily clinical setting or research. These results confirm that quality of life is significantly reduced in subjects with oropharyngeal dysphagia of different etiologies and severities. The data from the study demonstrated that the P-DHI may be a sensitive tool when attempting to identify the patient’s self-perception of the severity of their dysphagia. However, limitations of this tool will occur in the assessment of the illiterate Persian-speaking population in Iran. This limitation may be overcome through oral administration of the questions for the benefit of those who cannot read. The researchers must also be aware of differences in pronunciation and dialect if the instrument is to be administered orally. Our work was done in Zahedan and the Persian-language translation of the DHI proved valid and reliable for its use on Iranian individuals in Zahedan. However, to allow assessment of P-DHI of a wider range of individuals, future studies aimed at validating the scale in other city, culture and dialects should be encouraged. Also, there is an urgent need for future research to focus on evaluating the psychometric properties of other quality of life questionnaires in the Persian language.
